# Effects of precarious work on symptomatology of anxiety and depression in Chilean workers, a cross sectional study

**DOI:** 10.1186/s12889-021-10952-0

**Published:** 2021-05-17

**Authors:** Gonzalo Lopez, David Kriebel, Manuel Cifuentes, Margaret Quinn

**Affiliations:** 1 Work Environment Epidemiology, Department of Public Health, University of Massachusetts, One University Avenue, Lowell, 01854 MA USA; 2 Mariana de Osorio sin numero, Olmue, Region de Valparaiso Chile; 3 Department of Public Health, University of Massachusetts Lowell, One University Avenue, Lowell, 01854 MA USA; 4 Lowell Center for Sustainable Production, One University Avenue, Lowell, 01854 MA USA; 5 Public Health Program, Regis College, 235 Wellesley street, Weston, 02493 MA USA

**Keywords:** Precarious work, Anxiety, Depression, Neoliberalism

## Abstract

**Background:**

Precarious work is a broad definition for non-standard employment, often including unstable and insecure positions where workers permanently experience uncertainty; these types of jobs are growing steadily around the planet. Since the *coup d’état* in 1973, Chile has experienced a series of structural economic changes framed by neoliberal ideas cemented in the “Constitution of Pinochet.” Precarious work in Chile is a direct consequence of these ideas. This multidimensional phenomenon has progressively been entering employment areas where it was not previously present. As a result, there has been a rise in work precarization and its full impact on health is not well known. The goal of this study was to estimate the association of work precariousness with mental health outcomes in Chilean workers.

**Methods:**

Data were obtained from the Chilean Survey of Work and Health 2009–2010 (ENETS). Only valid records of salaried workers (excluding hourly-only or commission-only workers) in the private sector without missing values were included (*n* = 1900). After applying appropriate sampling weights, 1,461,727 workers were represented. Mental health was estimated as anxiety/depression levels using the 12-item General Health Questionnaire (GHQ-12). A multilevel multivariate generalized linear mixed model (GLMM) with negative binomial and log link distribution was used to study the association between precariousness and depression/anxiety.

**Results:**

Looking at the overall precariousness scale (range from zero to four), we observed an increase of approximately 34% in the depression/anxiety score (scale range from 0 to 36) for every unit on the precarious work overall scale (Relative Risk = 1.34, 95% CI = 1.28, 1.42) controlling for age, sex, and occupational group.

**Conclusion:**

Precarious work was associated with anxiety and depression as measured with the 12-item General Health Questionnaire. Controlling for demographic variables changed neither the direction nor the magnitude of the association.

**Supplementary Information:**

The online version contains supplementary material available at 10.1186/s12889-021-10952-0.

## Background

Precarious work is characterized by uncertainty and instability related to the ability to plan for the future; a precarious position is one in which the worker has a limited degree of autonomy or control of their destiny [1]. Precarious work may involve several dimensions of job insecurity, and not only the distinction between permanent and temporary employment. Precarious work has been growing steadily in the global market resulting in decreased fixed costs to employers and, at the same time, broadly decreased worker protections [2].

In Chile, the coup d’état of 1973 led to major social upheavals and government policies which transformed the country from an economic system based on strong social solidarity to a new model in which unregulated markets were designated as the main distribution mechanisms for goods and services [3]. Chile after the coup was quite explicitly seen as a neoliberal experiment [4–6] by the government, which introduced a series of political, economic, and social reforms [6] that profoundly impacted workers’ rights. Guided by the neoliberal economy doctrine of Milton Friedman and the Chicago School of Economics, precarious work became a cornerstone of the new economic order in Chile [4, 7]. These neoliberal policies were continued without major changes by consecutive governments long after the end of Pinochet’s dictatorship [8] [9].

During the last decades, precarious work also has grown rapidly and steadily across the world affecting nearly all developed and developing economies [10]. It is no longer only a feature of low-skill positions; and in so-called advanced economies like the U.S. and western Europe, many high-skill jobs are precarious [11], including those for journalists, university adjunct faculty, and other professionals who work as freelancers on a fee basis without hourly contracts or social security coverage [12].

Despite growing recognition in public health and the social sciences of the importance of studying the impacts of precarity on health and society, there is no consensus about an operational definition of precarious work. It is important to clarify that precarity is a characteristic of certain kinds of *work*, and not of the *workers* employed in those jobs. We often think of poor or disadvantaged people as being constrained to work under precarious conditions. But, as just noted, precarity is now widespread even in jobs requiring advanced degrees.

Precarious work is often defined in health and economic research using a dichotomous variable in which any type of job that does not offer permanent full employment is considered *precarious* [13, 14]. Less frequently, precarious work has been considered a multidimensional phenomenon independent from workers’ individual characteristics [15–17]. Our work builds on this latter approach.

Precarious work has been linked to a range of negative health impacts [10, 18], including stress and anxiety [15, 19, 20]. Chile, with nearly 50 years of neoliberal economic policies, has one of the highest prevalence rates of mental illness in the world [21] and mental health is the leading cause of medical leave [22]. Between 1990 and 2011, Chile experienced the second highest increase in suicides among the countries of the Organization for Economic Co-operation and Development (OECD), surpassed only by South Korea [23].

A comprehensive approach to understanding precarious work and developing an operational definition is needed to study the effects that this dynamic force exerts on workers’ health in general and mental health in particular. This study estimated the association between a multidimensional scale of exposure to precarious work and symptoms of anxiety and depression in Chilean workers.

## Methods

### Participants

The database used for this study was the Chilean Survey of Work and Health 2009–2010 (known by its Spanish acronym, ENETS). It contains multiple variables about employment and health conditions. The original sample (*n* = 9503) was representative of the entire adult Chilean population. ENETS was administered as an in-person confidential interview during the years 2009 and 2010 using a stratified random sampling with replacement. The sampling was designed to be representative of the entire population stratified at the regional level (Chile has 15 geo-political regions). The response rate was 79.3%. For this study, the sample had to be limited to salaried workers in the private sector because other survey participants were not asked a series of questions which were used to construct the precariousness scale (see below). In the Chilean context, as in the U.S., being a salaried employee means regularly receiving a predetermined amount of pay which does not vary based on the quality or quantity of work performed.

**Fig. 1:**
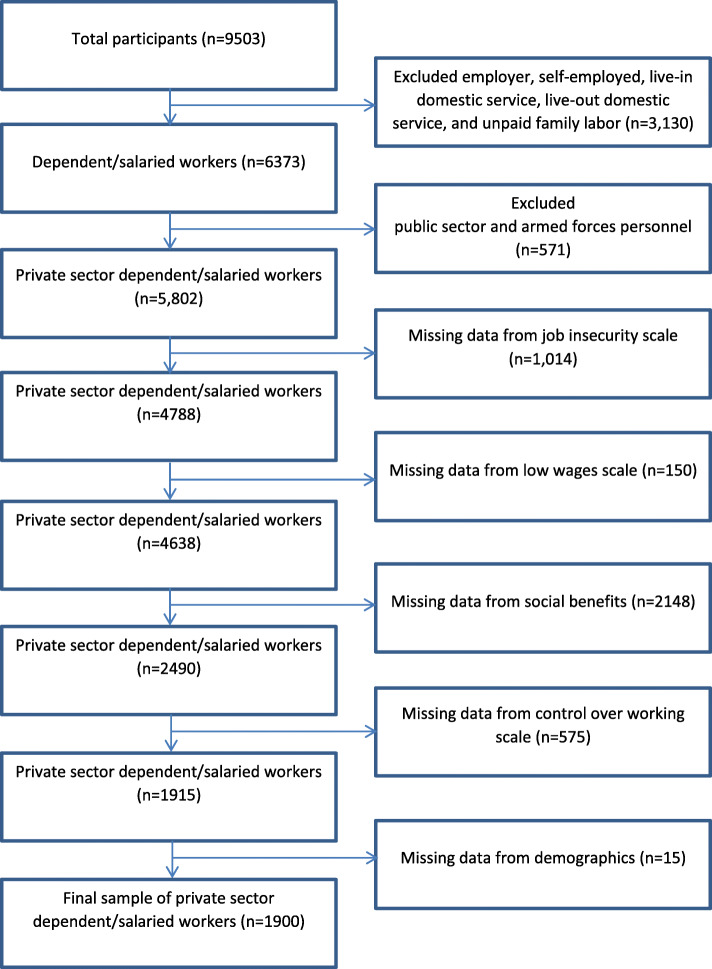
Flowchart of participants of the study

From the initial 9503 survey participants, 5802 were salaried workers in the private sector and therefore eligible to participate in the study (3701 were ineligible). Of these 5802, 3887 did not have complete information to compute some or all parts of the work precariousness scale and so were excluded. Incomplete demographic information led to 15 additional participants being dropped. This left 1900 participants with complete information on precariousness, demographics and the anxiety/depression score (GHQ-12) (see Fig. 1).

In Chile, the typical work arrangement is a regular monthly wage for up to 45 h per week distributed over 5 or 6 days with a limitation of no more than 10 h per day. Other arrangements are growing however, including part time and irregular hourly jobs. As noted, only salaried workers in the private sector and without any missing values in the variables of interest were included in this study, so that the proposed scale of precarious work could be fully evaluated. Compared to the full survey population, the selected group had a significantly higher proportion of males, urban residents, younger workers, and a higher educational level. There were no clear differences in terms of marital status and occupational groups (Table 1). After applying appropriate sampling weights, the included sample of 1900 represents 1,461,727 Chilean workers. An Institutional Review Board (IRB) approval was not necessary because the data are publicly available and de-identified.

**Table 1:** Demographic characteristics of included and excluded participants in the study population

Group	Ineligible (*n* = 3701)	Excluded for missing data (*n* = 3902)	Final sample (*n* = 1900)
**Sex**			
Male	2110 (57.0%)	2583 (66.2%)	1265 (66.5%)
Female	1591 (43.0%)	1319 (33.8%)	635 (33.4%)
**Residency**			
Urban	2923 (79.0%)	3246 (83.2%)	1677 (88.3%)
Rural	778 (21.0%)	656 (16.8%)	223 (11.7%)
**Groups of age**			
17–34 years old	640 (17.3%)	1431 (36.7%)	631 (33.2%)
35–50 years old	1511 (40.8%)	1545 (39.7%)	859 (45.2%)
51–69 years old	1370 (37.0%)	857 (22.0%)	400 (21.1%)
> 69 years old	179 (4.8%)	61 (1.6%)	10 (0.5%)
**Union**			
Unionized (Yes)	93 (16.3%)	422 (10.8%)	343 (18.1%)
Unionized (No)	475 (83.3%)	3464 (88.8%)	1556 (81.9%)
Dont answer	2 (0.40%)	6 (0.5%)	1 (0.1%)
**Marital status**			
Married	1808 (48.9%)	1644 (42.1%)	916 (48.2%)
Living together	530 (14.3%)	612 (15.7%)	331 (17.4%)
Annulled marriage	4 (0.1%)	5 (0.1%)	4 (0.2%)
Legally separated	128 (3.5%)	97 (2.5%)	56 (3.0%)
Not legally separated	222 (6.0%)	178 (4.6%)	95 (5.0%)
Widower/widow	178 (4.8%)	85 (2.2%)	26 (1.4%)
Single	807 (21.8%)	1270 (32.6%)	462 (24.3%)
Divorced	22 (0.6%)	10 (0.3%)	10 (0.5%)
**Educational level**			
Elementary school	1401 (38.7%)	1116 (29.1%)	395 (20.8%)
High school	1630 (45.0%)	2007 (52.4%)	1007 (53.0%)
Higher technical education	266 (7.3%)	329 (8.6%)	247 (13.0%)
University incomplete	108 (3.0%)	150 (3.9%)	76 (4.0%)
University complete	188 (5.2%)	215 (5.6%)	156 (8.2%)
University postgraduate	30 (0.8%)	15 (0.4%)	19 (1.0%)
**Occupational major groups**			
Legislator. senior officials and managers	147 (4.0%)	30 (0.8%)	29 (1.5%)
Professionals	156 (4.2%)	188 (4.8%)	142 (7.5%)
Technicians and associate professionals	255 (6.9%)	278 (7.1%)	230 (12.1%)
Clerks	100 (2.7%)	375 (9.6%)	210 (11.1%)
Service workers and shop and market sales workers	741 (20.0%)	646 (16.6%)	306 (16.1%)
Skilled agricultural and fishery workers	453 (12.2%)	260 (6.7%)	72 (3.8%)
Craft and related trades workers	603 (16.3%)	598 (15.3%)	299 (15.7%)
Plant and machine operators and assemblers	227 (6.1%)	521 (13.4%)	254 (13.4%)
Elementary occupations	984 (26.6%)	1006 (25.8%)	358 (18.8%)

### Independent variables

The predictor or independent variable is precarious work. To operationalize it, a scale was designed using the Delphi technique for expert consultation [24, 25]. Nine senior academic researchers with expertise in precarious work and mental health were recruited and agreed to participate. Each of them independently responded to a survey indicating which aspects of precarious work they considered most closely related to mental health outcomes. This was an iterative process, in which the participants converged through three rounds of increasingly narrowed selections, to select four relevant dimensions. Based on the answers obtained in the first step of the expert consultation, we reduced the list of aspects to those selected by at least one expert (Table 2). In the second step, we evaluated the expert rankings by frequency of agreement. Finally, we summarized the second-round expert judgments to produce a final list of the key dimension and components of precariousness.

**Table 2:** Summary of the second round of the expert consultation using three categories of response

Aspect of precarious work	Not important	Important	Very important	Total	Weighted average	Proportion of agreement
Job insecurity (lack of certainty about maintaining the current job in the future)	0	0	9	9	3.0	1.00
Low wages	0	1	8	9	2.9	0.78
Lack of social benefits (pension and/or health insurance)	0	2	7	9	2.8	0.61
Low control over work time (length of working time. Shift changes. Etc.)	1	1	7	9	2.7	0.58
Part-time work	3	5	1	9	1.8	0.36
Temporary and/or seasonal work	2	2	5	9	2.3	0.33
Self-employment	4	4	1	9	1.7	0.33
Short-term contract	2	5	2	9	2.0	0.33
Lack of collective bargaining	2	3	4	9	2.2	0.28
Lack of training	2	3	4	9	2.2	0.28
Indirect hire (use of intermediaries or contractors)	3	3	3	9	2.0	0.25
				K	0.13	12.9

**Fig. 2:**
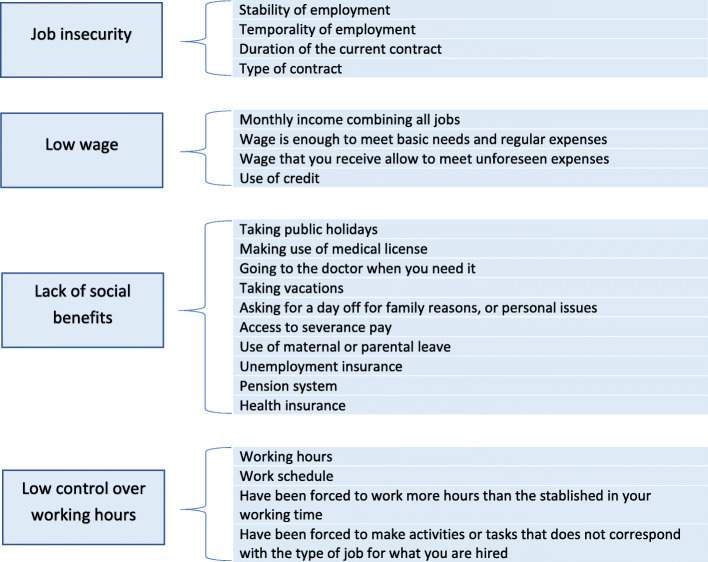
Dimension and variables of precarious work selected from the ENETS survey

Several questions from ENETS were selected to operationalize the concept of precarious work generated by the experts. The resulting scale is composed of the four dimensions selected by the panel of experts: 1) low job insecurity with four variables; 2) low wages with four variables; 3) lack of social security benefits with 10 variables; and 4) low control over working time with four variables (Fig. 2). Each dimension had scores from zero to one and the total scale had scores from zero to four. In the scale, a higher score represents an increased in precariousness. Additional details of the generation of this scale are available per request.

### Dependent variable

The General Health Questionnaire is a scale created in 1970 by Goldberg & Williams and includes a score measuring levels of anxiety and depression. It has been validated and used widely around the world [26] and was part of the ENETS questionnaire. It has 12 questions, each with ordinal answers with four choices. The individual scores were adjusted with a minimum of zero and a maximum of three, resulting in a scale range from zero to 36 points, where a higher score represents higher levels of depression/anxiety.

### Covariates and potential confounders

The dataset contained covariates sex, age, education level, region, marital status, occupation and unionization. Age, sex and occupation were retained in all models because they were considered a priori to be important covariates for understanding patterns of mental health, regardless of considerations of confounding. The other four covariates, with available data, education, region, marital status and unionization, were investigated as potential confounders due to their potential associations with either the independent variable work precariousness, the outcome anxiety/depression scores, or both. Each covariate’s bivariate associations with the independent and dependent variables were tested [27], and only those covariates with statistically significant associations (*p* < .05) with both the independent and dependent variables were retained as confounders (the “joint criteria” of confounding [28]).

#### Statistical analysis

Descriptive analysis considered central tendency and dispersion indicators for continuous variables and absolute and relative frequency (percentages) for discrete/categorical variables. Bivariate associations were performed using ANOVA, having as dependent variables anxiety/depression scores and work precariousness scale scores.

The GHQ-12 provides a zero to 36-count variable for the outcome; therefore, we used a generalized linear mixed model (GLMM) with negative binomial and log link distribution clustered by subject to control for the effect of sample weight on the standard errors. This model has zero as the lower limit of the distribution and is appropriate when there is over-dispersion of the outcome (variance larger than at least twice the mean). The regression coefficients were weighted by applying the expansion factor available in the ENETS database, in which every subject represented a group of people in their local area (as the original sample intended). Models were clustered by respondents to adjust standard errors for weights. All regression modeling used forward stepwise regression after having added the work precariousness score as the most important predictor variable. SPSS version 24 was used for all data management and analysis. Original database is available in Excel format in supplementary files.

## Results

### Anxiety/depression

For the Chilean workforce, the minimum value was 1 and the maximum 35. The overall mean was 10.1, with a standard deviation of 5.5. The mean score varied modestly by gender and urban/rural residence, while age had only a minimal effect (Table 3) and there was no association with unionization.

**Table 3:** Anxiety/depression and precarious work scores by demographics

Demographics	Anxiety/depression mean score (S.D)	Comparison of means for anxiety / depression (p value)	Precarious work scale mean score (S.D)	Comparison of means for precariousness scores (*p* value)	n (%)
**Sex**		**<.0001**		**.247**	
Men	9.5 (4.9)		1.33 (0.5)		1265 (66.6)
Women	11.2 (6.2)		1.36 (0.5)		635 (33.4)
**Geographical area**		**<.0001**		**.271**	
Urban	10.3 (5.6)		1.33 (0.5)		1677 (88.3)
Rural	8.7 (4.6)		1.38 (0.5)		223 (11.7)
**Age**		**<.0001**		**.351**	
17–34 years old	9.3 (5.1)		1.37 (0.5)		631 (33.2)
35–50 years old	10.7 (5.5)		1.33 (0.5)		859 (45.2)
51–69 years old	10.0 (5.7)		1.31 (0.5)		400 (21.1)
> 69 years old	10.3 (6.8)		1.34 (0.5)		10 (0.5)
**Unionization**		**.745**		**<.0001**	
Unionized (Yes)	10.2 (5.7)		1.23 (0.5)		344 (18.0)
Unionized (No)	10.1 (5.5)		1.37 (0.6)		1557 (81.9)
**Marital status**		**<.0001**		**.120**	
Married	9.9 (5.1)		1.30 (0.5)		915 (48.3)
Living together	10.0 (5.1)		1.40 (0.5)		330 (17.3)
Annulled marriage	7.5 (2.8)		1.19 (0.2)		4 (0.2)
Legally separated	12.1 (6.9)		1.46 (0.4)		56 (2.9)
Not legally separated	13.2 (6.9)		1.51 (0.5)		95 (5.0)
Widower/widow	14.3 (8.1)		1.42 (0.5)		26 (1.3)
Single	9.4 (5.2)		1.32 (0.5)		461 (24.3)
Divorced	14.8 (10)		1.46 (0.6)		10 (0.5)
**Educational level**		**<.0001**		**<.0001**	
Elementary school	10.4 (5.2)		1.5 (0.5)		385 (20.5)
High school	10.1 (5.6)		1.3 (0.5)		997 (53.1)
Higher technical education	9.6 (5.4)		1.1 (0.5)		246 (12.9)
University incomplete	9.5 (5.7)		1.1 (0.5)		76 (4.0)
University complete	10.0 (5.3)		1.0 (0.5)		156 (8.2)
University postgraduate	10.5 (6.0)		1.0 (0.5)		19 (1.0)
**Major Occupational Group**		**.108**		**<.0001**	
Legislator. senior officials and managers	8.7 (5.0)		0.78 (0.6)		29 (1.5)
Professionals	10.5 (5.5)		1.02 (0.5)		142 (7.5)
Technicians and associate professionals	9.7 (5.4)		1.20 (0.5)		230 (12.1)
Clerks	9.8 (5.3)		1.17 (0.5)		210 (11.0)
Service workers and shop and market sales workers	10.7 (5.7)		1.40 (0.5)		306 (16.1)
Skilled agricultural and fishery workers	9.7 (4.8)		1.31 (0.5)		72 (3.7)
Craft and related trades workers	9.9 (5.1)		1.43 (0.5)		299 (15.7)
Plant and machine operators and assemblers	9.8 (5.3)		1.39 (0.5)		254 (13.3)
Elementary occupations	10.5 (5.9)		1.56 (0.5)		358 (18.8)

### Precarious work

The minimum score for work precariousness was 0.0 and the maximum 3.35 out of a maximum of 4.0. The mean score was 1.3 and the standard deviation 0.58. Those participating in a union had a significantly lower score on work precariousness. No major occupational group had a score of zero (non-existent work precariousness). There was a clear inverse trend between socioeconomic status/education of the occupational group and precariousness scores, with the mean of Elementary Occupations like cleaning staff and temporary agricultural workers (simple and routine tasks which mainly require the use of hand-held tools and often some physical effort), being twice as large as the mean for Legislators, Senior Officials, and Managers (Table 3).

In general, all subscales of precarious work decreased with increased socioeconomic status of the occupational group. Plant and machine operators seem to be the exception with a low score in job security. (Table 4).

**Table 4:** Scores of precarious work subscales by occupation

	Dimensions of precarious work				Scale of precarious work
Occupation	Job insecurity (range 0–1)	Low wage	Lack of social security coverage	Low control over working time	Overall scale
Legislator. senior officials and managers	0.00	0.34	0.30	0.13	0.78
Professionals	0.04	0.47	0.32	0.25	1.09
Technicians and associate professionals	0.04	0.62	0.35	0.31	1.33
Clerks	0.02	0.64	0.36	0.32	1.35
Service workers and shop and market sales workers	0.04	0.67	0.46	0.40	1.58
Skilled agricultural and fishery workers	0.12	0.70	0.43	0.31	1.58
Craft and related trades workers	0.20	0.62	0.45	0.40	1.68
Plant and machine operators and assemblers	0.06	0.61	0.43	0.45	1.55
Elementary occupations	0.25	0.74	0.46	0.45	1.92

### Association of precariousness scales with anxiety/depression

Each subscale of work precariousness was evaluated for its association with anxiety/depression (Table 5). There was a modest 14% increase in symptoms of anxiety with a one-unit increase in the job insecurity subscale (relative risk = 1.14, 95% CI = 0.99, 1.31). All other scales had larger and more significant relative risks (RR = 1.54 for low wages; RR = 1.65 for lack of social security; and RR = 1.61 for low control over work time). Each subscale had a range from zero to one and, therefore, the relative risk can be understood as the ratio of increase in the anxiety/depression score by workers with, for example, no social benefits in comparison to workers with full social benefits.

**Table 5:** Associations of four subscales and overall scale of precarious work with anxiety/depression

	Association with anxiety/depression	
Subscales	Relative risk	95% CI
Job insecurity^a^	1.14	0.99–1.31
Low wages^a^	1.54	1.40–1.70
Lack of social security benefits^a^	1.65	1.48–1.85
Low control over work time^a^	1.61	1.39–1.87
**Overall precarious work scale**	1.35	1.28–1.42

^a^ Controlled by age, sex and occupational groups (10 categories)

In the overall precariousness scale, which has a range from zero to four (Table 5), we observed an increase in the anxiety/depression score of 35% (RR = 1.35; 95% CI 1.28, 1.42) for every unit increase on the precarious work scale. This would mean that in relation to workers with no precariousness at all, workers with the highest possible level of precariousness had a 5.40 times higher anxiety score (RR = 1.35 multiplied by 4).

### Analysis and impact of confounders

None of the four covariates education, region, marital status and unionization met the joint criteria to be classified as confounders [28] and so they were not retained. As noted, age, sex, and occupation were kept because they were considered a priori to be important covariates for understanding patterns of mental health, but excluding them had no important impacts on the model relative risks for the other covariates (data not shown).

## Discussion

In a study of salaried workers from the private sector, higher scores in a multidimensional scale of precarious work were associated with increased anxiety/depression scores. Like the entire scale, each work-precariousness subscale (job insecurity, low wages, lack of social security, and low control over working time) was associated with anxiety/depression. These results did not vary when controlling for potential confounders (sex, age, educational level, marital status, unionization, region and occupation).

The findings of the study are consistent with other studies on precarious work, which also found increased levels of anxiety and depression [14, 29, 30]. In our study, for each unit increase in the scale of precarious work, a 35% increase in the anxiety depression score was observed. This may be one reason for the poor mental health of Chilean workers, where the leading cause of medical leave is mental health problems, chiefly anxiety and depression [22].

The scale of precarious work developed within this research emphasized that precarity is a multi-dimensional phenomenon independent of the personal characteristics of the workers in a given job. The International Labor Organization (ILO) classifies precarious work based on employment status, considering full-time, permanent employment as non-precarious and all other arrangements (part-time, seasonal, casual, etc.) as precarious [31]. This study expanded that definition by including dimensions beyond full or part-time positions, including low wages, low control over working time, and lack of social benefits.

It is interesting to note that, of the four dimensions of our precarious work scale, job insecurity, often considered the defining characteristic had the weakest association with anxiety/depression. This underlines the importance of using a multidimensional scale; a dichotomous measure like the ILO definition, would have underestimated the strength of the association with anxiety/depression. It is also possible that not all relevant dimensions were captured with the work precariousness scale used in this study because we were constrained by the questions in the ENETS survey.

Broad occupational categories have often been considered proxy measures for socioeconomic status and education. In recent decades however, the diffusion of precarity up and down the ladder of occupations may undermine this common assumption about occupation and social class. Although the ANOVA test in Table 3 indicated that there were no significant differences in work precariousness scores among occupations, a clear gradient can be observed in which the occupations with higher SES had lower work precariousness scores. Periodic measurements with this new tool should permit the study of temporal trends and the impacts of policies affecting the labor market and the social contract.

One of the main limitations of this study was the reliance on a database that was not created to explore specifically the subject of this research. More appropriate questions need to be developed for a deeper exploration of precarious work and its evolving features, such as online platform work, the gig economy, and the increasing precariousness of very high-skill jobs. Additionally, and due to missing data, the sample size in this study was smaller than the total number of survey participants, diminishing the national workforce representativeness. However, we believe that it is likely that the group with higher precariousness and worse mental health would have had higher rates of missing responses, thus leading to an underestimation of the association under study.

An important strength of the study is that mental health status was self-reported, directly from ENETS, while work precariousness was obtained from multiple questions about objective descriptions of employment arrangements and working conditions. This should limit the strength of common methods bias. The same data capture process also excludes reverse causation, unless the presence of high anxiety/depression scores makes workers self-select into more precarious jobs. This interpretation is not supported by the fact that compared with other Organization for Economic Co-operation and Development (OECD) countries, Chile’s indicators of mental health have worsened at an accelerated rate [32]. We conclude, therefore, that reverse causation and inflated results due to common method bias are unlikely to be serious limitations.

## Conclusion

We conclude that the steady acceptance of precarious work as a normalized work arrangement is likely to have deleterious impacts on social life, and on the relationships that workers have with their jobs.

A labor market consequence (work precariousness) of macroeconomic decisions (market liberalization) can be measured and evaluated for its impacts on mental health (anxiety/depression).

A valid multidimensional tool measuring work precariousness is important as societies search for economic systems that can provide better and more equitable living conditions. When planning the organization of work, the items and subscales of the multidimensional work precariousness tool may provide stakeholders with useful language with which to discuss policies and actions to improve work and reduce exposures to hazardous conditions.

Almost 50 years after the 1973 coup in Chile, which brought neo-liberal economic policies and a major shift towards precarious work, the country is currently in the midst of another major upheaval out of which will come a new constitution. We hope that this research can contribute in some small way to improving the conditions of work in the new Chile now in process.

## Supplementary Information

**Additional file 1.**

## Data Availability

The dataset used in this article was gathered and published by the Department of Epidemiology of the Minister of Health of Chile. It is legally publically available. Unfortunately, the COVID-19 pandemic has led to interruptions in access to various government websites, including the one where public datasets including this one are made available. The authors have also made the data available at: https://drive.google.com/file/d/1rhxH9QifXmUhHVWNrqTJdzBS7QsqKEri/view?usp=sharing

## Abbreviations

ENETS: Chilean Survey of Work and Health 2009–2010
GHQ-12: 12 Item General Health Questionnaire
GLMM: Generalized Linear Mixed Model
OECD: Organization for Economic Co-operation and Development
IRB: Institutional Review Board
ILO: International Labour Organization
